# A Domino Oxidation/Arylation/Protodecarboxylation Reaction of Salicylaldehydes: Expanded Access to *meta*‐Arylphenols

**DOI:** 10.1002/asia.201500506

**Published:** 2015-06-25

**Authors:** Junfei Luo, Sara Preciado, Solomon Olatokunbo Araromi, Igor Larrosa

**Affiliations:** ^1^ School of ChemistryUniversity of ManchesterOxford RoadManchesterM13 9PLUK; ^2^ School of Biological and Chemical ScienceQueen Mary University of LondonMile End RoadLondonE1 4NSUK

**Keywords:** arylation, C−H activation, *meta*-selective, phenols, salicylaldehydes

## Abstract

A method that allows salicylaldehydes to be efficiently transformed into *meta*‐arylated phenol derivatives through a cascade oxidation/arylation/protodecarboxylation sequence is presented. We demonstrate that the aldehyde functional group can be used as a convenient removable directing group to control site selectivity in C−H activation. Aldehydes are easily introduced into the starting materials and the group is readily cleaved after the C−H functionalization event.

Transition metal catalyzed C−H activation provides an atom‐ and step‐economical alternative to the formation of C−C, C−X, C−O, and C−N bonds.^1^ Site‐selective functionalization of ubiquitous C−H bonds is a highly attractive strategy as it allows efficient and reliable access to target molecules. There have been tremendous advances in this field, in particular for the functionalization of aromatic compounds.^2^ In these cases, a regioselective controlling group is usually required owing to the similar reactivity of the C−H bonds in benzene derivatives. The most popular strategy is the use of directing groups, which offer site‐selective functionalization through chelation assistance, and dozens of methods for *ortho*‐functionalization based on this strategy have been reported in recent years.^3^, ^4^ However, it becomes more challenging to apply this type of strategy when the target C−H bond is distant from the directing group; the development of direct *meta*‐selective functionalization remains a significant challenge.^5^ Additionally, if a directing group functionality is not present in the starting material, further synthetic steps are required to install, and possibly remove, the directing group, which is a substantial drawback for synthetic applications. Significant research effort has therefore been focused on the development of directing groups that are easily introduced and removed, and so are applicable to the synthesis of a wider range of target molecules. We recently developed a novel strategy using a transient carboxyl group that facilitates a palladium‐catalyzed cross‐coupling reaction *ortho‐* to the carboxylic acid (*meta*‐ to the hydroxyl substituent) which, followed by decarboxylation of the arene, affords *meta*‐arylated phenol derivatives (Scheme 1 a).^6^, ^7^ This method allowed the synthesis of *meta*‐arylphenols from cheap phenols in a single synthetic operational step, and substituents including electron‐donating and electron‐withdrawing groups are tolerated. However, the carboxyl group is installed by a Kolbe–Schmitt carboxylation, requiring high CO_2_ pressure (20–100 atm) and high temperature (120–300 °C),^8^ which can limit the synthetic applications of the method.

**Scheme 1:**
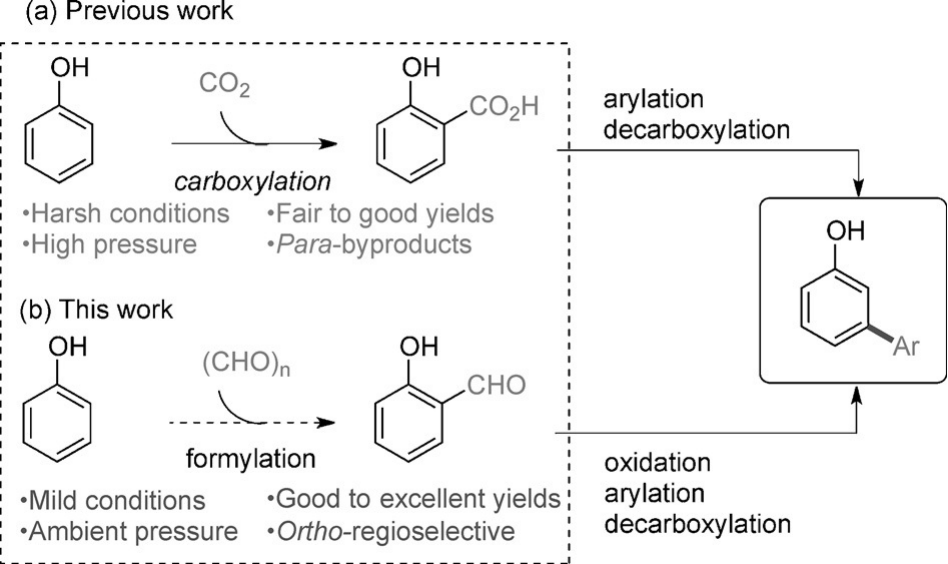
Traceless directing groups lead to *meta*‐arylphenols.

To avoid the use of harsh conditions, it is key to find a removable directing group that: (1) can be easily installed under mild conditions; (2) can facilitate an *ortho*‐functionalization; (3) can be easily cleaved, revealing the desired *meta*‐arylated products. Salicylaldehyde motifs are widely found in natural products as well as being key precursors to a variety of useful molecules.^9^ Importantly, the aldehyde group can be easily and efficiently introduced into phenol derivatives under mild conditions by a simple *ortho*‐selective formylation (Scheme 1 b).^10^, ^11^ However, due to its sensitivity under common C−H arylation reaction conditions, the aldehyde group has rarely been used as a directing group before.^12^ In this report, we describe how aldehydes can be used as efficient traceless directing groups, allowing the synthesis of *meta*‐arylated phenol derivatives under a palladium‐catalyzed system.

As a starting point, the reaction between salicylaldehyde (**1 a**) and 1‐iodo‐3,5‐dimethylbenzene (**2 a**) catalyzed by Pd(OAc)_2_ (2 mol %) in the presence of Ag_2_CO_3_ (0.5 equiv) in AcOH, heating at 150 °C for 16 h was studied (Table 1, entry 1). Interestingly, 17 % of the desired product was obtained. The amount of Ag_2_CO_3_ proved crucial, and 1.0 equivalent of Ag_2_CO_3_ performed best (Table 1, entry 2). The source of the Pd catalyst was found to be important, with PEPPSI‐IPr (from the pyridine enhanced precatalyst preparation stabilization and initiation family) leading to the best yield (Table 1, entry 4). Moreover, the addition of K_2_CO_3_ significantly improved the yields (Table 1, entries 5 and 7). Finally, increasing the amount of Pd to 5 mol % led to 72 % yield (Table 1, entry 7). Further efforts aiming to facilitate the oxidation of the formyl group in situ by the addition of stronger oxidants, were unsuccessful. It was envisaged that oxidation to salicylic acid may be required before *ortho*‐metalation.^13^ However, the addition of *p*‐benzoquinone and *m*‐CPBA (*m*‐chloroperoxybenzoic acid) completely shut down the reaction (Table 1, entries 8 and 9).

**Table 1:** Optimization of *meta*‐arylation from salicylaldehyde.^[a]^

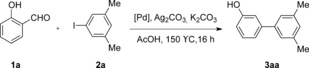				
Entries^[a]^	Pd (mol %)	Ag_2_CO_3_ [equiv]	K_2_CO_3_ [equiv]	Yield of **3 aa** [%]
1	Pd(OAc)_2_ (2)	0.5	0	17
2	Pd(OAc)_2_ (2)	1.0	0	27
3	Pd(OAc)_2_ (2)	1.5	0	18
4	PEPPSI‐IPr (2)	1.0	0	35
5	PEPPSI‐IPr (2)	1.0	0.5	54
6	PEPPSI‐IPr (2)	1.0	2.0	60
7	PEPPSI‐IPr (5)	1.0	2.0	72
8^[b]^	PEPPSI‐IPr (2)	1.0	2.0	0
9^[c]^	PEPPSI‐IPr (2)	1.0	2.0	0

[a] Unless otherwise noted, all the reactions were carried out using 0.25 mmol of **1 a** and 0.75 mmol of **2 a**. Yields were determined by ^1^H NMR analysis using 1,3,5‐trimethoxybenzene as an internal standard. [b] 1.0 equiv of *p*‐benzoquinone was added into the reaction. [c] 1.0 equiv of *m*‐CPBA was added into the reaction.

With suitable conditions in hand, we first examined different substitution patterns on the aryl iodide (Scheme 2). Gratifyingly, both electron‐donating (**3 aa**, **3 ad**, and **3 ah**) and electron‐withdrawing groups (**3 ab**, **3 ac**, **3 ae**, **3 af**, **3 ai**–**al**) were tolerated at *meta*‐ and *para*‐positions, affording moderate yields. However, only 21 % of the desired product was achieved when *para*‐iodoanisole was used (**3 ag**), with 53 % product of arylation at the aldehyde C−H bond instead.^14^, ^15^ *ortho*‐Substituents at the iodoarene are not tolerated, potentially due to the increased steric hindrance.^16^ Substituted salicylaldehydes containing both electron‐donating (**3 ea**, **3 fa**, **3 ja**, and **3 ka**) and electron‐withdrawing groups (**3 ba**–**da**, **3 ga**–**ia**) substituted at the 3‐ and 4‐positions were found to be suitable, and disubstituted salicylaldehyde (**3 ma**) was obtained in fair yield. However, low yield was obtained when the strongly electron‐donating methoxy group was present (**3 la**): in this case, 3‐methoxyphenol was observed after formal protodecarbonylation of starting material. Similarly to the lack of tolerance for *ortho*‐substituted iodoarenes, 5‐substituted salicylaldehydes showed no reactivity (Scheme 3).^16^

**Scheme 2:**
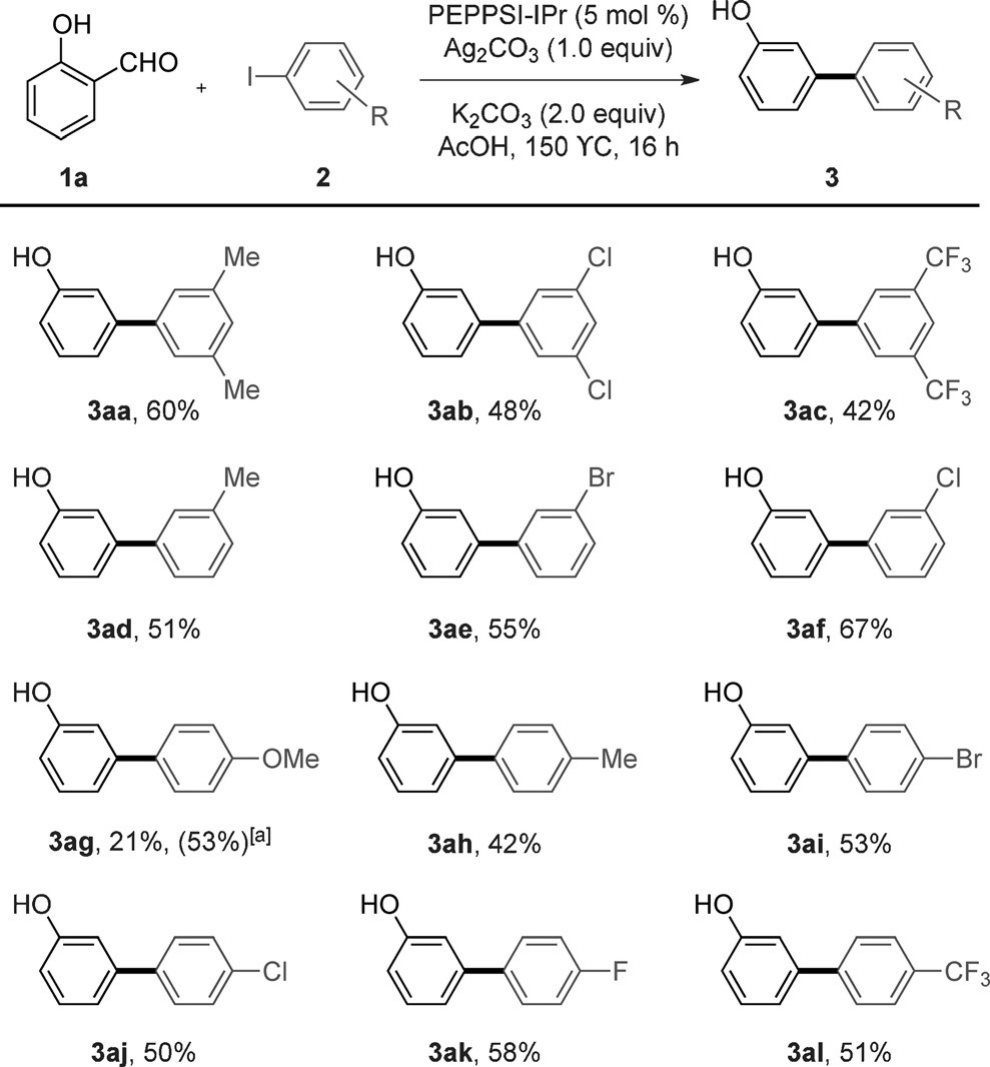
Scope of the domino oxidation/arylation/protodecarboxylation process on substituted iodoarenes. 3.0 equiv of **2** with respect to **1 a** were used. Yields are of isolated pure material. [a] Isolated yield of side arylation product at the aldehyde C−H bond.

**Scheme 3:**
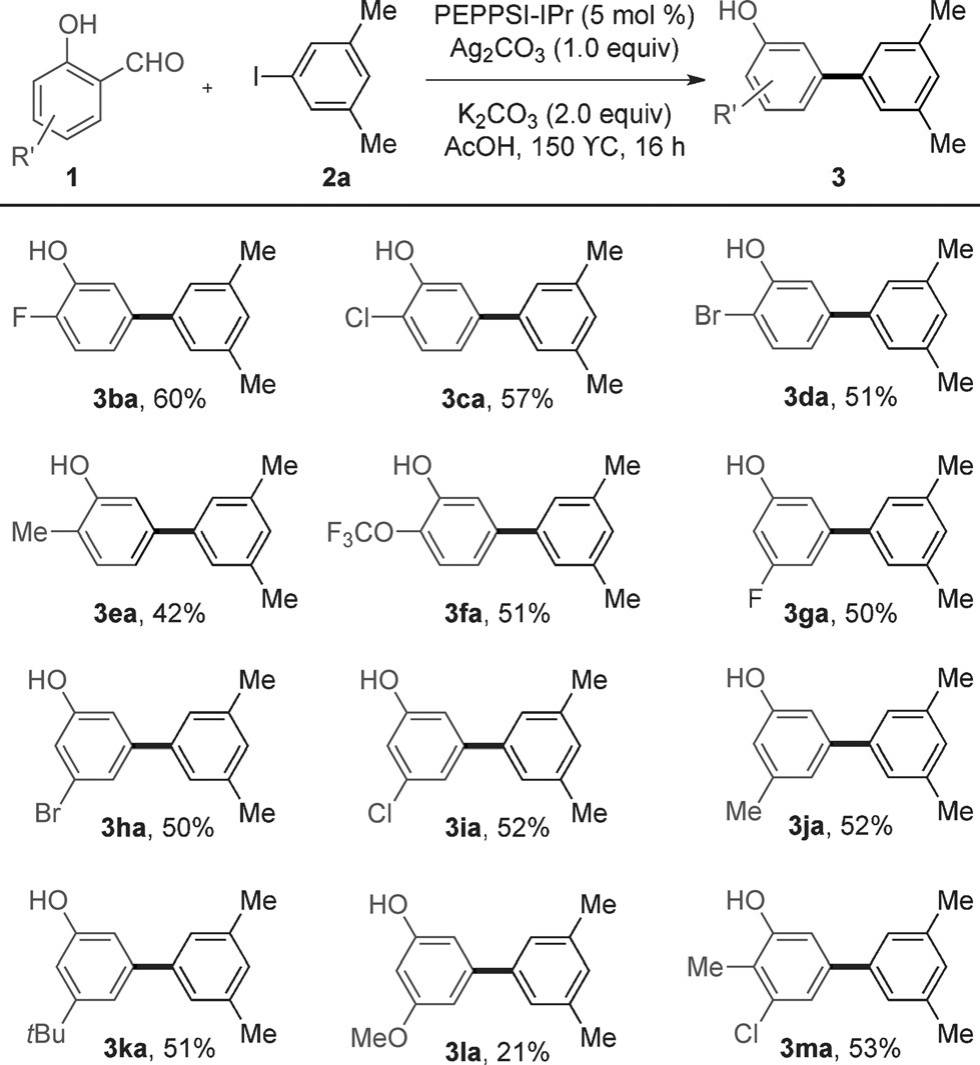
Scope of the domino oxidation/arylation/protodecarboxylation process on salicylaldehydes. 3.0 equiv of **2 a** with respect to **1** were used.

We set out to examine how this transformation of salicylaldehydes to *meta*‐arylphenols was occurring. A few examples of the use of the aldehyde group as an *ortho*‐directing group under Pd‐catalyzed conditions have been reported.12a,12b Furthermore, Pd‐catalyzed decarbonylation of aldehydes has also been shown by Maiti and co‐workers.^17^ Additionally, given that Tollens’ reagent is known to oxidize an aldehyde into the corresponding carboxylic acid, we postulated that the salicylaldehyde could be oxidized to salicylic acid in the presence of Ag_2_CO_3_. Based on the above, we speculated that the transformation could occur through three possible pathways (Scheme 4): (1) *ortho*‐Arylation of salicylaldehyde, followed by a palladium catalyzed decarbonylation, affording the *meta*‐arylphenol. (2) *ortho*‐Arylation of salicylaldehyde, followed by oxidation of the arylated salicylaldehyde intermediate to the corresponding acid, with protodecarboxylation generating *meta*‐arylated phenol. (3) Oxidation of the salicylaldehyde starting material to salicylic acid, with arylation and protodecarboxylation of salicylic acid proceeding to *meta*‐arylphenol.^13^ The examination of the crude reaction mixtures revealed that no *ortho*‐arylated salicylaldehyde intermediate was observed, with 20 % of salicylic acid being obtained instead (Scheme 5 a), thus indicating that pathway three may be in operation. To probe this mechanism further, we heated salicylaldehyde in the presence of Ag_2_CO_3_ but only starting materials were recovered (Scheme 5 b). However, 42 % of salicylic acid was obtained when salicylaldehyde was submitted to the standard arylation conditions in the absence of iodoarene. A Wacker‐type oxidation process might be the explanation of this Pd‐catalyzed oxidation of salicylaldehyde.^18^ Finally, the use of the iodide abstractor NMe_4_OAc, which has been shown to be an efficient replacement for AgOAc when oxidation is not required,^19^ resulted in no arylation reaction. These experiments suggest that this transformation of salicylaldehydes to *meta*‐arylated phenol derivatives proceeds through an oxidation/arylation/proto‐decarboxylation process.

**Scheme 4:**
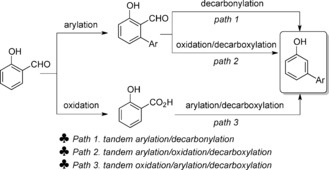
Possible reaction pathways.

**Scheme 5:**
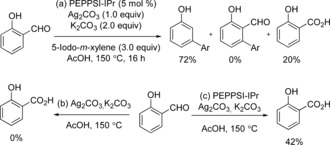
Investigation of reactions.

In summary, we have described a method for the preparation of *meta*‐arylated phenols from salicylaldehyde derivatives. Importantly, unlike most directing groups, the aldehyde group can be easily introduced into the starting material and removed after C−H functionalization in a tandem fashion. A preliminary mechanistic study indicates that the reaction occurs through three steps involving a palladium‐catalyzed oxidation of the salicylaldehyde followed by *ortho*‐arylation of the salicylic acid intermediate and subsequent protodecarboxylation of the arylated product. Remarkably, these three steps occur in a cascade process, allowing for a simple and efficient synthetic operation.

## Experimental Section

**General Information**

All chemicals used in this work were obtained from commercial sources and used without further purification. Salicylaldehyde starting materials were prepared by Skattebø’s procedure11b,11f except **1 a** and **1 e**, which were purchased from Sigma Aldrich. Analytical thin‐layer chromatography was performed on pre‐coated Merck silica gel F254 plates and visualized under UV light. Melting points were obtained using a Bibby Stuart Scientific apparatus and are uncorrected. IR spectra were recorded using a Bruker Tensor 37 FTIR machine and are quoted in cm^−1^. ^1^H NMR spectra, recorded at 400 MHz, are referenced to the residual solvent peak at 7.26 ppm (CDCl_3_). ^13^C NMR spectra, recorded at 101 MHz, are referenced to the residual solvent peak at 77.0 ppm (CDCl_3_).

**General Procedure**: A mixture of **1** (0.25 mmol), **2** (3.0 equiv), PEPPSI‐IPr (5 mol %), Ag_2_CO_3_ (1.0 equiv, and K_2_CO_3_ (2.0 equiv), in AcOH (0.5 mL) was heated in a sealed vial at 150 °C for 16 h. After this time, the reaction mixture was filtered through a plug of Celite with EtOAc (4×5 mL). The filtrate was evaporated to dryness. The crude product was purified by column chromatography (hexanes/EtOAc 90:10). The characterization data of compounds **3** are shown in the Supporting Information.

## Supporting information

As a service to our authors and readers, this journal provides supporting information supplied by the authors. Such materials are peer reviewed and may be re‐organized for online delivery, but are not copy‐edited or typeset. Technical support issues arising from supporting information (other than missing files) should be addressed to the authors.

Supplementary
