# Supplementary material for: A Domino Oxidation/Arylation/Protodecarboxylation Reaction of Salicylaldehydes: Expanded Access to meta‐Arylphenols
Source: Chem Asian J. 2015 Jun 25;11(3):347–50. doi: 10.1002/asia.201500506 (PMC4744983; doi:10.1002/asia.201500506)
Supplement: Supplementary file 1 — Supplementary [file ASIA-11-347-s001.pdf]

CHEMISTRY
AN ASIAN JOURNAL

Supporting Information

A Domino Oxidation/Arylation/Protodecarboxylation Reaction of Salicylaldehydes: Expanded Access to *meta*-Arylphenols

Junfei Luo,[a, b] Sara Preciado,[b] Solomon Olatokunbo Araromi,[b] and Igor Larrosa*[a]

asia_201500506_sm_miscellaneous_information.pdf

Table of Contents

General experimental information. *Page S2*

Procedures and characterization data. *Page S3*

References. *Page S14*

Spectroscopic data. *Page S15*

General experimental information

All chemicals used in this work were obtained from commercial sources and used without further purification. Salicylaldehydes starting materials were prepared by Skattebø's procedure[1,2] except salicylaldehyde and 3-methylsalicylaldehyde that were purchased from Sigma Aldrich. Analytical thin-layer chromatography was performed on pre-coated Merk silica gel F254 plates and visualized under a UV light. Melting points were obtained using a Bibby Stuart Scientific apparatus and are uncorrected. IR spectra were recorded using a Bruker Tensor 37 FTIR machine and are quoted in cm^{-1}. ^{1}H NMR spectra, recorded at 400 MHz, are referenced to the residual solvent peak at 7.26 ppm ($CDCl_3$). ^{13}C NMR spectra, recorded at 101 MHz, are referenced to the residual solvent peak at 77.0 ppm ($CDCl_3$).

Procedures and characterization data

3',5'-Dimethyl-[1,1'-biphenyl]-3-ol

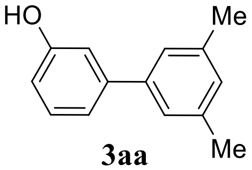

3aa

A mixture of PEPPSI-IPr (8.5 mg, 0.0125 mmol), Ag_2CO_3 (69.0 mg, 0.25 mmol), K_2CO_3 (69.0 mg, 0.50 mmol), salicylaldehyde (27 µL, 0.25 mmol) and 1-iodo-3,5-dimethylbenzene (110 µL, 0.75 mmol) in AcOH (500 µL) was heated at 150 °C for 16 h. After this time, the reaction mixture was filtered through a plug of Celite® with EtOAc (4 × 5 mL). The filtrate was evaporated to dryness. The crude product was purified by column chromatography (Hexanes:EtOAc 90:10) to afford 3',5'-dimethyl-[1,1'-biphenyl]-3-ol (**3aa**) as a light orange oil (29.7 mg, 60%). **^{1}H NMR** (400 MHz, $CDCl_3$) δ 7.29 (t, *J* = 7.9 Hz, 1H), 7.20 (s, 2H), 7.17-7.15 (m, 1H), 7.07-7.05 (m, 1H), 7.01 (s, 1H), 6.80 (ddd, *J* = 8.0, 2.6, 0.9 Hz, 1H), 4.76 (s, 1H), 2.38 (s, 6H) ppm. **^{13}C NMR** (101 MHz, $CDCl_3$) δ 155.7, 143.3, 140.8, 138.3, 129.9, 129.1, 125.1, 119.9, 114.1, 114.0, 21.4 ppm. These data are consistent with those previously reported.[3]

3',5'-dichloro-[1,1'-biphenyl]-3-ol

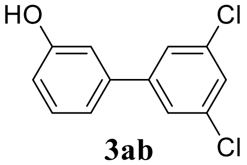

3ab

A mixture of PEPPSI-IPr (8.5 mg, 0.0125 mmol), Ag_2CO_3 (69.0 mg, 0.25 mmol), K_2CO_3 (69.0 mg, 0.50 mmol), salicylaldehyde (27 µL, 0.25 mmol) and 3,5-dichloroiodobenzene (205.0 mg, 0.75 mmol) in AcOH (500 µL) was heated at 150 °C for 16 h. After this time, the reaction mixture was filtered through a plug of Celite® with EtOAc (4 × 5 mL). The filtrate was evaporated to dryness. The crude product was purified by column chromatography (Hexanes:EtOAc 90:10) to afford 3',5'-dichloro-[1,1'-biphenyl]-3-ol (**3ab**) as an off white solid (28.5 mg, 48%). **mp**: 88-91 °C. **IR**: 3228.5, 1556.8, 1402.9, 1203.4, 768.6 cm^{-1}. **^{1}H NMR** (400 MHz, $CDCl_3$) δ 7.43 (d, *J* = 1.9 Hz, 2H), 7.34-7.30 (m, 2H), 7.10 (ddd, *J* = 7.7, 1.7, 0.9 Hz, 1H), 7.01-7.00 (m, 1H), 6.87 (ddd, *J* = 8.1, 2.5, 0.9 Hz, 1H), 4.90 (s, 1H). ppm. **^{13}C NMR** (101 MHz, $CDCl_3$) δ 156.0, 143.7, 140.3, 135.3, 130.3, 127.4, 125.7, 119.7, 115.4, 114.1.ppm. **HRMS**: calcd for $C_{12}H_9Cl_2O$, 239.0025 ($M+H^+$); found, 239.0025.

3',5'-Bis(trifluoromethyl)-[1,1'-biphenyl]-3-ol

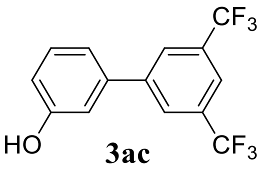

3ac

A mixture of PEPPSI-IPr (8.5 mg, 0.0125 mmol), Ag_2CO_3 (69.0 mg, 0.25 mmol), K_2CO_3 (69.0 mg, 0.50 mmol), salicylaldehyde (27 µL, 0.25 mmol) and 1-iodo-3,5-bis(trifluoromethyl)benzene (133 µL, 0.75 mmol) in AcOH (500 µL) was heated at 150 ºC for 16 h. After this time, the reaction mixture was filtered through a plug of Celite® with EtOAc (4 × 5 mL). The filtrate was evaporated to dryness. The crude product was purified by column chromatography (Hexanes:EtOAc 85:15) to afford 3',5'-bis(trifluoromethyl)-[1,1'-biphenyl]-3-ol (**3ac**) as a white solid (32.0mg, 42%). **^{1}H NMR** (400 MHz, $CDCl_3$) δ 7.99 (s, 2H), 7.86 (s, 1H), 7.38 (t, *J* = 7.9 Hz, 1H), 7.18 (ddd, *J* = 7.7, 1.6, 0.9 Hz, 1H), 7.08 (t, *J* = 2.4 Hz, 1H), 6.92 (ddd, *J* = 8.1, 2.5, 0.8 Hz, 1H), 4.96 (s, 1H) ppm. **^{13}C NMR** (101 MHz, $CDCl_3$) δ 156.2, 142.8, 140.0, 132.1 (q, *J* = 33.2 Hz), 130.6, 127.2 (d, *J* = 2.7 Hz), 123.4 (q, *J* = 272.7 Hz), 121.1 (dt, *J* = 7.8, 3.8 Hz), 119.8, 115.8, 114.2 ppm. These data are consistent with those previously reported.[4]

3'-Methyl-[1,1'-biphenyl]-3-ol

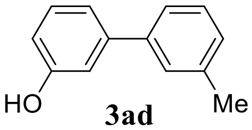

3ad

A mixture of PEPPSI-IPr (8.5 mg, 0.0125 mmol), Ag_2CO_3 (69.0 mg, 0.25 mmol), K_2CO_3 (69.0 mg, 0.50 mmol), salicylaldehyde (27 µL, 0.25 mmol) and 3-iodotoluene (97 µL, 0.75 mmol) in AcOH (500 µL) was heated at 150 ºC for 16 h. After this time, the reaction mixture was filtered through a plug of Celite® with EtOAc (4 × 5 mL). The filtrate was evaporated to dryness. The crude product was purified by column chromatography (Hexanes:EtOAc 90:10) to afford 3'-methyl-[1,1'-biphenyl]-3-ol (**3ad**) as an orange oil (23.4 mg, 51%). **^{1}H NMR** (400 MHz, $CDCl_3$) δ 7.40-7.28 (m, 4H), 7.18-7.16 (m, 2H), 7.07-7.06 (m, 1H), 6.82 (ddd, *J* = 8.0, 2.6, 0.9 Hz, 1H), 4.84 (s, 1H), 2.42 (s, 3H) ppm. **^{13}C NMR** (101 MHz, $CDCl_3$) δ 155.8, 143.2, 140.7, 138.4, 129.9, 128.7, 128.3, 127.9, 124.2, 119.8, 114.1, 114.1, 21.5 ppm. These data are consistent with those previously reported.[3]

3'-bromo-[1,1'-biphenyl]-3-ol

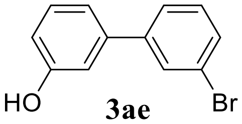

3ae

A mixture of PEPPSI-IPr (6.8 mg, 0.01 mmol), Ag_2CO_3 (55.0 mg, 0.2 mmol), K_2CO_3 (55.0 mg, 0.40 mmol), salicylaldehyde (22 µL, 0.20 mmol) and 3-bromoiodobenzene (76 µL, 0.60 mmol) in AcOH (500 µL) was heated at 150 ºC for 16 h. After this time, the reaction mixture was filtered through a plug of Celite® with EtOAc (4 × 5 mL). The filtrate was evaporated to dryness. The crude product was purified by column chromatography (Hexanes:EtOAc 90:10) to afford 3'-bromo-[1,1'-biphenyl]-3-ol (**3ae**) as a brownish oil (27.4 mg, 55%). **IR**: 3315.4, 1557.5, 1466.1, 1194.1, 771.9, 687.8 cm^{-1}. **^{1}H NMR** (400 MHz, $CDCl_3$) δ 7.71 (t, *J* = 1.7 Hz, 1H), 7.50-7.47 (m, 2H), 7.32 (t, *J* = 8.0 Hz, 1H), 7.30 (app t, *J* = 8.1 Hz, 1H), 7.13 (d, *J* = 7.7 Hz, 1H), 7.03-7.02 (m, 1H), 6.86-6.83 (m, 1H), 4.81 (s, 1H) ppm. **^{13}C NMR** (101 MHz, $CDCl_3$) δ 155.9, 142.9, 141.5, 130.4, 130.3, 130.26, 130.21, 125.7, 122.9, 119.8, 114.8, 114.1 ppm. **HRMS**: calcd for $C_{12}H_{10}BrO$, 248.9910 ($M+H^+$); found, 248.9910.

3'-Chloro-[1,1'-biphenyl]-3-ol

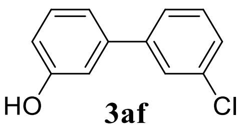

3af

A mixture of PEPPSI-IPr (8.5 mg, 0.0125 mmol), Ag_2CO_3 (69.0 mg, 0.25 mmol), K_2CO_3 (69.0 mg, 0.50 mmol), salicylaldehyde (27 µL, 0.25 mmol) and 3-chloroiodobenzene (179.0 mg, 0.75 mmol) in AcOH (500 µL) was heated at 150 ºC for 16 h. After this time, the reaction mixture was filtered through a plug of Celite® with EtOAc (4 × 5 mL). The filtrate was evaporated to dryness. The crude product was purified by column chromatography (Hexanes:EtOAc 90:10) to afford 3'-methyl-[1,1'-biphenyl]-3-ol (**3af**) as a brownish oil (34.5 mg, 67%). **IR**: 3324.9, 1564.2, 1196.2, 774.0, 688.5 cm^{-1}. ^{1}**H NMR** (400 MHz, $CDCl_3$) δ 7.56-7.55 (m, 1H), 7.44 (dt, *J* = 7.4, 1.6 Hz, 1H), 7.38-7.30 (m, 3H), 7.14 (ddd, *J* = 7.7, 1.7, 0.9 Hz, 1H), 7.04-7.03 (m, 1H), 6.84 (ddd, *J* = 8.1, 2.5, 0.9 Hz, 1H), 4.79 (s, 1H) ppm. ^{13}**C NMR** (101 MHz, $CDCl_3$) δ 155.9, 142.6, 141.6, 134.7, 130.2, 130.0, 127.5, 127.3, 125.3, 119.8, 114.8, 114.1 ppm. **HRMS**: calcd for $C_{12}H_{10}ClO$, 205.0342 ($M+H^+$); found, 205.0415.

4'-Methoxy-[1,1'-biphenyl]-3-ol

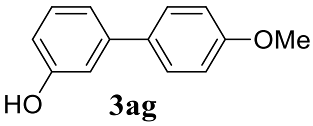

3ag

A mixture of PEPPSI-IPr (8.5 mg, 0.0125 mmol), Ag_2CO_3 (69.0 mg, 0.25 mmol), K_2CO_3 (69.0 mg, 0.50 mmol), salicylaldehyde (27 µL, 0.25 mmol) and 4-iodoanisole (176.0 mg, 0.75 mmol) in AcOH (500 µL) was heated at 150 ºC for 16 h. After this time, the reaction mixture was filtered through a plug of Celite® with EtOAc (4 × 5 mL). The filtrate was evaporated to dryness. The crude product was purified by column chromatography (Hexanes:EtOAc 90:10) to afford 4'-methoxy-[1,1'-biphenyl]-3-ol (**3ag**) as an off white solid (10.6 mg, 21%). ^{1}**H NMR** (400 MHz, $CDCl_3$) δ 7.53-7.49 (m, 2H), 7.28 (t, *J* = 8.0 Hz, 1H), 7.13 (ddd, *J* = 7.7, 1.6, 1.0 Hz, 1H), 7.03-7.02 (m, 1H), 6.99-6.95 (m, 2H), 6.78 (ddd, *J* = 8.0, 2.5, 0.9 Hz, 1H), 4.78 (s, 1H), 3.85 (s, 3H) ppm. ^{13}**C NMR** (101 MHz, $CDCl_3$) δ 159.3, 155.8, 142.6, 133.3, 129.9, 128.1, 119.4, 114.2, 113.7, 113.6, 55.4 ppm. These data are consistent with those previously reported.[3]

Aldehyde arylation side product: (2-hydroxyphenyl)-(4-methoxyphenyl)methanone

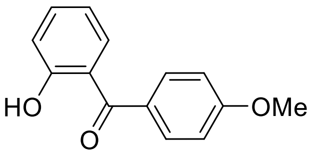

A mixture of PEPPSI-IPr (8.5 mg, 0.0125 mmol), Ag_2CO_3 (69.0 mg, 0.25 mmol), K_2CO_3 (69.0 mg, 0.50 mmol), salicylaldehyde (27 µL, 0.25 mmol) and 4-iodoanisole (176.0 mg, 0.75 mmol) in AcOH (500 µL) was heated at 150 ºC for 16 h. After this time, the reaction mixture was filtered through a plug of Celite® with EtOAc (4 × 5 mL). The filtrate was evaporated to dryness. The crude product was purified by column chromatography (Hexanes:EtOAc 95:05) to afford (2-hydroxyphenyl)-(4-methoxyphenyl)methanone as a light orange solid (30.2 mg, 53%). ^{1}**H NMR** (400 MHz, $CDCl_3$) δ

11.99 (s, 1H), 7.72 (d, J = 8.7 Hz, 2H), 7.63 (d, J = 7.9 Hz, 1H), 7.50 (t, J = 7.2 Hz, 1H), 7.07 (d, J = 8.3 Hz, 1H), 7.00 (d, J = 8.7 Hz, 2H), 6.89 (t, J = 7.5 Hz, 1H), 3.90 (s, 3H) ppm. **^{13}C NMR** (101 MHz, $CDCl_3$) δ 200.1, 162.9 (×2), 135.9, 133.3, 131.9, 130.3, 119.4, 118.6, 118.4, 113.7, 55.6 ppm. These data are consistent with those previously reported.[5]

4'-Methyl-[1,1'-biphenyl]-3-ol

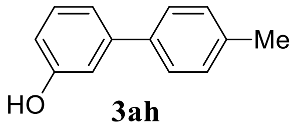

3ah

A mixture of PEPPSI-IPr (8.5 mg, 0.0125 mmol), Ag_2CO_3 (69.0 mg, 0.25 mmol), K_2CO_3 (69.0 mg, 0.50 mmol), salicylaldehyde (27 µL, 0.25 mmol) and 4-iodotoluene (164.0 mg, 0.75 mmol) in AcOH (500 µL) was heated at 150 ºC for 16 h. After this time, the reaction mixture was filtered through a plug of Celite® with EtOAc (4 × 5 mL). The filtrate was evaporated to dryness. The crude product was purified by column chromatography (Hexanes:EtOAc 90:10) to afford 4'-methyl-[1,1'-biphenyl]-3-ol (**3ah**) as a brownish solid (19.3 mg, 42%). **IR**: 3280.9, 2915.4, 1588.7, 1189.0, 772.5 cm^{-1}. **^{1}H NMR** (400 MHz, $CDCl_3$) δ 7.49-7.46 (m, 2H), 7.30 (app t, J = 7.9 Hz, 1H), 7.25 (app dd, J = 8.4, 0.6 Hz, 2H), 7.17 (ddd, J = 7.7, 1.7, 1.0 Hz, 1H), 7.05 (dd, J = 2.5, 1.7 Hz, 1H), 6.80 (ddd, J = 8.0, 2.5, 1.0 Hz, 1H), 4.85 (s, 1H), 2.40 (s, 3H) ppm. **^{13}C NMR** (101 MHz, $CDCl_3$) δ 155.8, 143.0, 137.9, 137.3, 130.0, 129.5, 127.0, 119.6, 113.9 (×2), 21.1 ppm. **MS (EI)**: *m/z* (M^+), 184.0. These data are consistent with those previously reported.[6]

4'-Bromo-[1,1'-biphenyl]-3-ol

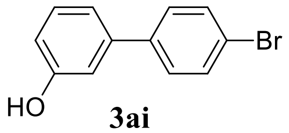

3ai

A mixture of PEPPSI-IPr (6.8 mg, 0.01 mmol), Ag_2CO_3 (55.0 mg, 0.2 mmol), K_2CO_3 (55.0 mg, 0.40 mmol), salicylaldehyde (22 µL, 0.20 mmol) and 1-bromo-4-iodobenzene (170.0 mg, 0.6 mmol) in AcOH (500 µL) was heated at 150 ºC for 16 h. After this time, the reaction mixture was filtered through a plug of Celite® with EtOAc (4 × 5 mL). The filtrate was evaporated to dryness. The crude product was purified by column chromatography (Hexanes:EtOAc 90:10) to afford 4'-bromo-[1,1'-biphenyl]-3-ol (**3ai**) as a light orange solid (26.2 mg, 53%). **^{1}H NMR** (400 MHz, $CDCl_3$) δ 7.58-7.54 (m, 2H), 7.45-7.42 (m, 2H), 7.31 (t, J = 7.9 Hz, 1H), 7.13 (ddd, J = 7.7, 1.6, 0.9 Hz, 1H), 7.03-7.02 (m, 1H), 6.83 (ddd, J = 8.1, 2.5, 0.9 Hz, 1H), 4.79 (s, 1H) ppm. **^{13}C NMR** (101 MHz, $CDCl_3$) δ 156.1, 141.9, 139.8, 132.0, 130.3, 128.8, 121.9, 119.7, 114.7, 114.1 ppm. These data are consistent with those previously reported.[3]

4'-Chloro-[1,1'-biphenyl]-3-ol

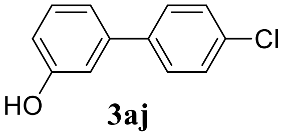

3aj

A mixture of PEPPSI-IPr (6.8 mg, 0.01 mmol), Ag_2CO_3 (55.0 mg, 0.2 mmol), K_2CO_3 (55.0 mg, 0.40 mmol), salicylaldehyde (22 µL, 0.20 mmol) and 1-chloro-4-iodobenzene (143.0 mg, 0.6 mmol) in AcOH (500 µL) was heated at 150 ºC for 16 h. After this time, the reaction mixture was filtered through a plug of Celite® with EtOAc (4 × 5 mL). The filtrate was evaporated to dryness. The crude product was purified by column chromatography (Hexanes:EtOAc 90:10) to afford 4'-chloro-[1,1'-biphenyl]-3-ol (**3aj**) as a light orange solid (20.3 mg, 50%). **^{1}H NMR** (400 MHz, $CDCl_3$) δ 7.51-7.48 (m, 2H), 7.41-7.38 (m, 2H), 7.31 (t, *J* = 7.9 Hz, 1H), 7.13 (ddd, *J* = 7.7, 1.6, 0.9 Hz, 1H), 7.03-7.02 (m, 1H), 6.83 (ddd, *J* = 8.1, 2.5, 0.9 Hz, 1H), 4.85 (s, 1H) ppm. **^{13}C NMR** (101 MHz, $CDCl_3$) δ 156.0, 141.8, 139.2, 133.6, 130.1, 128.9, 128.4, 119.6, 114.5, 114.0 ppm. These data are consistent with those previously reported.[3]

4'-Fluoro-[1,1'-biphenyl]-3-ol

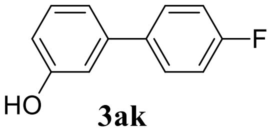

3ak

A mixture of PEPPSI-IPr (6.8 mg, 0.01 mmol), Ag_2CO_3 (55.0 mg, 0.2 mmol), K_2CO_3 (55.0 mg, 0.40 mmol), salicylaldehyde (22 µL, 0.20 mmol) and 1-fluoro-4-iodobenzene (69 µL, 0.6 mmol) in AcOH (500 µL) was heated at 150 ºC for 16 h. After this time, the reaction mixture was filtered through a plug of Celite® with EtOAc (4 × 5 mL). The filtrate was evaporated to dryness. The crude product was purified by column chromatography (Hexanes:EtOAc 90:10) to afford 4'-fluoro-[1,1'-biphenyl]-3-ol (**3ak**) as an off white solid (21.9 mg, 58%). **^{1}H NMR** (400 MHz, $CDCl_3$) δ 7.56-7.50 (m, 2H), 7.30 (t, *J* = 7.9 Hz, 1H), 7.15-7.09 (m, 3H), 7.02-7.01 (m, 1H), 6.82 (ddd, *J* = 8.1, 2.5, 0.9 Hz, 1H), 4.83 (bs, 1H) ppm. **^{13}C NMR** (101 MHz, $CDCl_3$) δ 162.6 (d, *J* = 246.6 Hz), 155.9, 142.1, 136.9 (d, *J* = 3.2 Hz), 130.1, 128.7, 128.6, 119.7, 115.6 (d, *J* = 21.4 Hz), 114.1 (d, *J* = 18.4 Hz) ppm. These data are consistent with those previously reported.[3]

4'-(Trifluoromethyl)-[1,1'-biphenyl]-3-ol

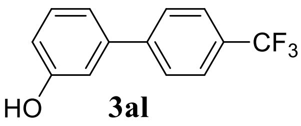

3al

A mixture of PEPPSI-IPr (8.5 mg, 0.0125 mmol), Ag_2CO_3 (69.0 mg, 0.25 mmol), K_2CO_3 (69.0 mg, 0.50 mmol), salicylaldehyde (27 µL, 0.25 mmol) and 4-iodobenzotrifluoride (110 µL, 0.75 mmol) in AcOH (500 µL) was heated at 150 ºC for 16 h. After this time, the reaction mixture was filtered through a plug of Celite® with EtOAc (4 × 5 mL). The filtrate was evaporated to dryness. The crude product was purified by column chromatography (Hexanes:EtOAc 90:10) to afford 4'-

(trifluoromethyl)-[1,1'-biphenyl]-3-ol (**3al**) as a pale yellow solid (30.2 mg, 51%). **^{1}H NMR** (400 MHz, $CDCl_3$) δ 7.73-7.68 (m, 4H), 7.37 (t, *J* = 7.9 Hz, 1H), 7.21 (ddd, *J* = 7.7, 1.6, 0.9 Hz, 1H), 7.11-7.10 (m, 1H), 6.91 (ddd, *J* = 8.1, 2.5, 0.9 Hz, 1H), 4.91 (s, 1H) ppm. **^{13}C NMR** (101 MHz, $CDCl_3$) δ 156.0, 144.2, 141.6, 130.3, 129.6 (q, *J* = 32.5 Hz), 124.3 (q, *J* = 272.0 Hz), 127.4, 125.7 (q, *J* = 3.8 Hz), 119.9, 115.1, 114.3 ppm. These data are consistent with those previously reported.[4]

4-Fluoro-3',5'-dimethyl-[1,1'-biphenyl]-3-ol

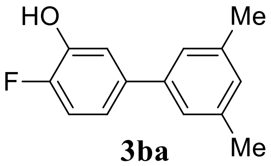

3ba

A mixture of PEPPSI-IPr (8.5 mg, 0.0125 mmol), Ag_2CO_3 (69.0 mg, 0.25 mmol), K_2CO_3 (69.0 mg, 0.50 mmol), 3-fluorosalicylaldehyde (35.0 mg, 0.25 mmol) and 1-iodo-3,5-dimethylbenzene (110 µL, 0.75 mmol) in AcOH (500 µL) was heated at 150 ºC for 16 h. After this time, the reaction mixture was filtered through a plug of Celite® with EtOAc (4 × 5 mL). The filtrate was evaporated to dryness. The crude product was purified by column chromatography (Hexanes:EtOAc 90:10) to afford 4-fluoro-3',5'-dimethyl-[1,1'-biphenyl]-3-ol (**3ba**) as a light orange oil (32.2 mg, 60%). **^{1}H NMR** (400 MHz, $CDCl_3$) δ 7.22 (dd, *J* = 8.5, 2.2 Hz, 1H), 7.15 (s, 2H), 7.14-7.09 (m, 1H), 7.06-7.04 (m, 1H), 6.99 (s, 1H), 5.17 (s, 1H), 2.37 (s, 6H) ppm. **^{13}C NMR** (101 MHz, $CDCl_3$) δ 150.6 (d, *J* = 237.4 Hz), 143.5 (d, *J* = 14.6 Hz), 140.1, 138.7 (d, *J* = 3.6 Hz), 138.3, 129.1, 125.0, 119.5 (d, *J* = 6.4 Hz), 116.0 (d, *J* = 1.8 Hz), 115.5 (d, *J* = 18.3 Hz), 21.4 ppm. These data are consistent with those previously reported.[3]

4-Chloro-3',5'-dimethyl-[1,1'-biphenyl]-3-ol

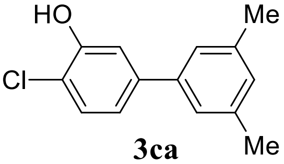

3ca

A mixture of PEPPSI-IPr (8.5 mg, 0.0125 mmol), Ag_2CO_3 (69.0 mg, 0.25 mmol), K_2CO_3 (69.0 mg, 0.50 mmol), 3-chlorosalicylaldehyde (39.1 mg, 0.25 mmol) and 1-iodo-3,5-dimethylbenzene (110 µL, 0.75 mmol) in AcOH (500 µL) was heated at 150 ºC for 16 h. After this time, the reaction mixture was filtered through a plug of Celite® with EtOAc (4 × 5 mL). The filtrate was evaporated to dryness. The crude product was purified by column chromatography (Hexanes:EtOAc 90:10) to afford 4-chloro-3',5'-dimethyl-[1,1'-biphenyl]-3-ol (**3ca**) as a light yellow solid (33.4 mg, 57%). **mp**: 32-35 °C. **IR**: 3517.5, 3025.9, 2916.7, 1571.8, 1201.4, 1179.8, 1045.2, 848.7, 807.8 cm^{-1}. **^{1}H NMR** (400 MHz, $CDCl_3$) δ 7.35 (d, *J* = 8.3 Hz, 1H), 7.24 (d, *J* = 2.1 Hz, 1H), 7.17 (s, 2H), 7.09 (dd, *J* = 8.3, 2.1 Hz, 1H), 7.01 (s, 1H), 5.54 (s, 1H) 2.38 (s, 6H) ppm. **^{13}C NMR** (101 MHz, $CDCl_3$) δ 151.4, 142.1, 139.8, 138.4, 129.4, 129.0, 124.9, 120.2, 118.7, 114.8, 21.4 ppm. **MS** (**EI**): m/z (M+), 232.1.

4-Bromo-3',5'-dimethyl-[1,1'-biphenyl]-3-ol

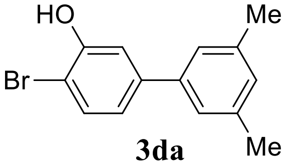

3da

A mixture of PEPPSI-IPr (6.8 mg, 0.01 mmol), Ag_2CO_3 (55.0 mg, 0.2 mmol), K_2CO_3 (55.0 mg, 0.40 mmol), 3-bromo-2-hydroxybenzaldehyde (40.2 mg, 0.20 mmol) and 1-iodo-3,5-dimethylbenzene (110 µL, 0.75 mmol) in AcOH (500 µL) was heated at 150 ºC for 16 h. After this time, the reaction mixture was filtered through a plug of Celite® with EtOAc (4 × 5 mL). The filtrate was evaporated to dryness. The crude product was purified by column chromatography (Hexanes:EtOAc 90:10) afford 4-bromo-3',5'-dimethyl-[1,1'-biphenyl]-3-ol (**3da**) as a colorless oil (28.4 mg, 51%). ^{1}**H NMR** (400 MHz, $CDCl_3$) δ 7.49 (d, *J* = 8.3 Hz, 1H), 7.24 (d, *J* = 2.1 Hz, 1H), 7.17 (s, 2H), 7.05-7.01 (m, 2H), 5.52 (s, 1H), 2.38 (s, 6H) ppm. ^{13}**C NMR** (101 MHz, $CDCl_3$) δ 152.4, 143.0, 139.8, 138.4, 132.0, 129.5, 124.9, 120.7, 114.7, 109.0, 21.4 ppm. These data are consistent with those previously reported.[3]

3',4,5'-Trimethyl-[1,1'-biphenyl]-3-ol

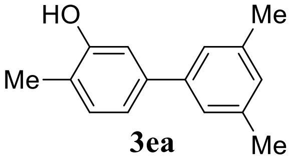

3ea

A mixture of PEPPSI-IPr (6.8 mg, 0.01 mmol), Ag_2CO_3 (55.0 mg, 0.2 mmol), K_2CO_3 (55.0 mg, 0.40 mmol), 2-hydroxy-3-methylbenzaldehyde (25 µL, 0.20 mmol) and 1-iodo-3,5-dimethylbenzene (110 µL, 0.75 mmol) in AcOH (500 µL) was heated at 150 ºC for 16 h. After this time, the reaction mixture was filtered through a plug of Celite® with EtOAc (4 × 5 mL). The filtrate was evaporated to dryness. The crude product was purified by column chromatography (Hexanes:EtOAc 90:10) to afford 3',4,5'-trimethyl-[1,1'-biphenyl]-3-ol (**3ea**) as an orange oil (17.9 mg, 42%). ^{1}**H NMR** (400 MHz, $CDCl_3$) δ 7.18-7.16 (m, 3H), 7.08 (dd, *J* = 7.7, 1.7 Hz, 1H), 7.01-7.00 (m, 1H), 6.98 (s, 1H), 4.69 (s, 1H), 2.37 (s, 6H), 2.29 (s, 3 H) ppm. ^{13}**C NMR** (101 MHz, $CDCl_3$) δ 153.9, 140.8, 140.8, 138.2, 131.2, 128.8, 124.9, 122.5, 119.6, 113.7, 21.4, 15.4 ppm. These data are consistent with those previously reported.[3]

3',5'-Dimethyl-4-(trifluoromethoxy)-[1,1'-biphenyl]-3-ol

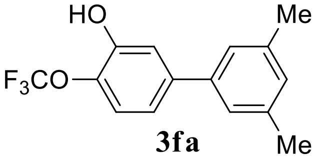

3fa

A mixture of PEPPSI-IPr (8.5 mg, 0.0125 mmol), Ag_2CO_3 (69.0 mg, 0.25 mmol), K_2CO_3 (69.0 mg, 0.50 mmol), 2-hydroxy-3-(trifluoromethoxy)benzaldehyde (51.5 mg, 0.25 mmol) and 1-iodo-3,5-dimethylbenzene (110 µL, 0.75 mmol) in AcOH (500 µL) was heated at 150 ºC for 16 h. After this time, the reaction mixture was filtered through a plug of Celite® with EtOAc (4 × 5 mL). The filtrate was evaporated to dryness. The crude product was purified by column chromatography

(Hexanes:EtOAc 90:10) to afford 3',5'-dimethyl-4-(trifluoromethoxy)-[1,1'-biphenyl]-3-ol (**3fa**) as a colorless oil (35.9 mg, 51%). **^{1}H NMR** (400 MHz, $CDCl_3$) δ 7.28-7.25 (m, 2H), 7.17 (s, 2H), 7.12 (dd, *J* = 8.5, 2.2 Hz, 1H), 7.01 (s, 1H), 5.54 (s, 1H), 2.38 (s, 6H) ppm. **^{13}C NMR** (101 MHz, $CDCl_3$) δ 147.8, 141.8, 139.7, 138.4, 135.7 (d, *J* = 1.5 Hz), 129.5, 125.0, 121.4 (d, *J* = 0.9 Hz), 120.81 (q, *J* = 258.9 Hz), 119.6, 116.0, 21.4 ppm. These data are consistent with those previously reported.[3]

5-Fluoro-3',5'-dimethyl-[1,1'-biphenyl]-3-ol

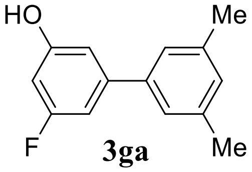

3ga

A mixture of PEPPSI-IPr (8.5 mg, 0.0125 mmol), Ag_2CO_3 (69.0 mg, 0.25 mmol), K_2CO_3 (69.0 mg, 0.50 mmol), 4-fluorosalicylaldehyde (39.0 mg, 0.25 mmol) and 1-iodo-3,5-dimethylbenzene (110 µL, 0.75 mmol) in AcOH (500 µL) was heated at 150 °C for 16 h. After this time, the reaction mixture was filtered through a plug of Celite® with EtOAc (4 × 5 mL). The filtrate was evaporated to dryness. The crude product was purified by column chromatography (Hexanes:EtOAc 90:10) to afford 5-fluoro-3',5'-dimethyl-[1,1'-biphenyl]-3-ol (**3ga**) as a brownish orange solid (27.1 mg, 50%). **^{1}H NMR** (400 MHz, $CDCl_3$) δ 7.16-7.15 (m, 2H), 7.02-7.01 (m, 1H), 6.87 (ddd, *J* = 9.8, 2.3, 1.5 Hz, 1H), 6.83-6.82 (m, 1H), 6.54 (dt, *J* = 9.8, 2.3 Hz, 1H), 4.90 (s, 1H), 2.37 (s, 6H) ppm. **^{13}C NMR** (101 MHz, $CDCl_3$) δ 163.8 (d, *J* = 244.9 Hz), 156.8 (d, *J* = 12.0 Hz), 144.6 (d, *J* = 9.8 Hz), 139.7 (d, *J* = 2.6 Hz), 138.4, 129.7, 124.9, 109.9 (d, *J* = 2.7 Hz), 106.7 (d, *J* = 22.3 Hz), 101.8 (d, *J* = 24.9 Hz), 21.36 ppm. These data are consistent with those previously reported.[4]

5-Bromo-3',5'-dimethyl-[1,1'-biphenyl]-3-ol

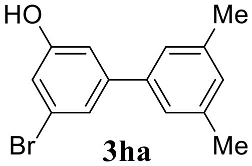

3ha

A mixture of PEPPSI-IPr (8.5 mg, 0.0125 mmol), Ag_2CO_3 (69.0 mg, 0.25 mmol), K_2CO_3 (69.0 mg, 0.50 mmol), 4-bromosalicylaldehyde (50.3 mg, 0.25 mmol) and 1-iodo-3,5-dimethylbenzene (110 µL, 0.75 mmol) in AcOH (500 µL) was heated at 150 °C for 16 h. After this time, the reaction mixture was filtered through a plug of Celite® with EtOAc (4 × 5 mL). The filtrate was evaporated to dryness. The crude product was purified by column chromatography (Hexanes:EtOAc 90:10) to afford 5-bromo-3',5'-dimethyl-[1,1'-biphenyl]-3-ol (**3ha**) as a light yellow solid (34.6 mg, 50%). **^{1}H NMR** (400 MHz, $CDCl_3$) δ 7.30 (app. t, *J* = 1.6 Hz, 1H), 7.15 (s, 2H), 7.02 (s, 1H), 6.97 (app. p, *J* = 2.3 Hz, 2H), 4.83 (s, 1H), 2.37 (s, 6H) ppm. **^{13}C NMR** (101 MHz, $CDCl_3$) δ 156.4, 144.7, 139.4, 138.4, 129.7, 125.0, 123.0 (CH+C), 117.3, 113.1, 21.4 ppm. These data are consistent with those previously reported.[3]

5-Chloro-3',5'-dimethyl-[1,1'-biphenyl]-3-ol

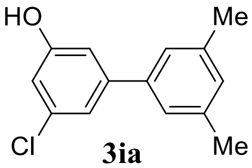

3ia

A mixture of PEPPSI-IPr (8.5 mg, 0.0125 mmol), Ag_2CO_3 (69.0 mg, 0.25 mmol), K_2CO_3 (69.0 mg, 0.50 mmol), 4-chlorosalicylaldehyde (39.1 mg, 0.25 mmol) and 1-iodo-3,5-dimethylbenzene (110 µL, 0.75 mmol) in AcOH (500 µL) was heated at 150 ºC for 16 h. After this time, the reaction mixture was filtered through a plug of Celite® with EtOAc (4 × 5 mL). The filtrate was evaporated to dryness. The crude product was purified by column chromatography (Hexanes:EtOAc 90:10) to afford 5-chloro-3',5'-dimethyl-[1,1'-biphenyl]-3-ol (**3ia**) as a light orange solid (30.0 mg, 52%). **^{1}H NMR** (400 MHz, $CDCl_3$) δ 7.15-7.14 (m, 3H), 7.02 (s, 1H), 6.93-6.92 (m, 1H), 6.82-6.81 (m, 1H), 4.84 (s, 1H), 2.37 (s, 6H) ppm. **^{13}C NMR** (101 MHz, $CDCl_3$) δ 156.3, 144.4, 139.5, 138.4, 135.1, 129.7, 124.9, 120.1, 114.4, 112.6, 21.3 ppm. **HRMS**: calcd for $C_{14}H_{13}ClO$, 232.0655 (M^+); found, 232.0649. These data are consistent with those previously reported.[3]

3',5,5'-Trimethyl-[1,1'-biphenyl]-3-ol

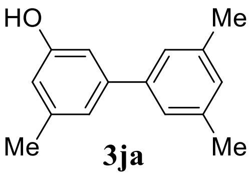

3ja

A mixture of PEPPSI-IPr (8.5 mg, 0.0125 mmol), Ag_2CO_3 (69.0 mg, 0.25 mmol), K_2CO_3 (69.0 mg, 0.50 mmol), 4-methylsalicylaldehyde (34.0 mg, 0.25 mmol) and 1-iodo-3,5-dimethylbenzene (110 µL, 0.75 mmol) in AcOH (500 µL) was heated at 150 ºC for 16 h. After this time, the reaction mixture was filtered through a plug of Celite® with EtOAc (4 × 5 mL). The filtrate was evaporated to dryness. The crude product was purified by column chromatography (Hexanes:EtOAc 90:10) to afford 3',5,5'-trimethyl-[1,1'-biphenyl]-3-ol (**3ja**) as a light orange solid (27.7 mg, 52%). **^{1}H NMR** (400 MHz, $CDCl_3$) δ 7.18 (s, 2H), 6.99-6.97 (m, 2H), 6.85-6.84 (m, 1H), 6.63 (s, 1H), 4.66 (s, 1H), 2.37 (s, 6H), 2.36 (s, 3H) ppm. **^{13}C NMR** (101 MHz, $CDCl_3$) δ 155.7, 143.1, 140.9, 139.9, 138.2, 129.0, 125.0, 120.8, 114.8, 111.3, 21.4, 21.4 ppm. These data are consistent with those previously reported.[3]

5-(*tert*-Butyl)-3',5'-dimethyl-[1,1'-biphenyl]-3-ol

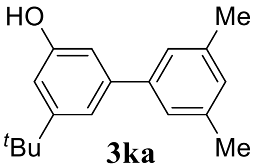

3ka

A mixture of PEPPSI-IPr (8.5 mg, 0.0125 mmol), Ag_2CO_3 (69.0 mg, 0.25 mmol), K_2CO_3 (69.0 mg, 0.50 mmol), 4-*tert*-butyl-2-hydroxybenzaldehyde (44.6 mg, 0.25 mmol) and 1-iodo-3,5-dimethylbenzene (110 µL, 0.75 mmol) in AcOH (500 µL) was heated at 150 ºC for 16 h. After this

time, the reaction mixture was filtered through a plug of Celite® with EtOAc (4 × 5 mL). The filtrate was evaporated to dryness. The crude product was purified by column chromatography (Hexanes:EtOAc 90:10) to afford 5-(*tert*-butyl)-3',5'-dimethyl-[1,1'-biphenyl]-3-ol (**3ka**) as an orange oil (32.6 mg, 51%). **^{1}H NMR** (400 MHz, $CDCl_3$) δ 7.17 (s, 2H), 7.16 (app. t, J = 1.6 Hz, 1H), 7.00 (s, 1H), 6.85 (m, 2H), 4.70 (s, 1H), 2.38 (s, 6H), 1.35 (s, 9H) ppm. **^{13}C NMR** (101 MHz, $CDCl_3$) δ 155.5, 153.5, 142.9, 141.5, 138.2, 129.0, 125.2, 117.2, 111.4, 111.3, 34.86, 31.4, 21.4 ppm. **HRMS**: calcd for $C_{18}H_{22}O$, 255.1671 ($M+H^+$); found, 255.1743. These data are consistent with those previously reported.[4]

5-Methoxy-3',5'-dimethyl-[1,1'-biphenyl]-3-ol

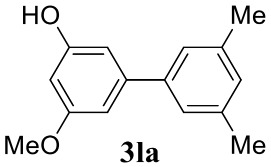

3la

A mixture of PEPPSI-IPr (8.5 mg, 0.0125 mmol), Ag_2CO_3 (69.0 mg, 0.25 mmol), K_2CO_3 (69.0 mg, 0.50 mmol), 4-methoxysalicylaldehyde (38.0 mg, 0.25 mmol) and 1-iodo-3,5-dimethylbenzene (110 µL, 0.75 mmol) in AcOH (500 µL) was heated at 150 ºC for 16 h. After this time, the reaction mixture was filtered through a plug of Celite® with EtOAc (4 × 5 mL). The filtrate was evaporated to dryness. The crude product was purified by column chromatography (Hexanes:EtOAc 90:10) to afford 5-methoxy-3',5'-dimethyl-[1,1'-biphenyl]-3-ol (**3la**) as a brownish oil (11.8 mg, 21%). **^{1}H NMR** (400 MHz, $CDCl_3$) δ 7.17 (s, 2H), 7.00 (s, 1H), 6.71-6.70 (m, 1H), 6.65-6.64 (m, 1H), 6.39 (app. t, J = 2.3 Hz, 1H), 4.83 (s, 1H), 3.84 (s, 3H), 2.37 (s, 6H) ppm. **^{13}C NMR** (101 MHz, $CDCl_3$) δ 161.1, 156.8, 144.1, 140.8, 138.2, 129.3, 125.0, 106.9, 105.7, 100.3, 55.4, 21.4 ppm. These data are consistent with those previously reported.[3]

5-Chloro-3',4,5'-trimethyl-[1,1'-biphenyl]-3-ol

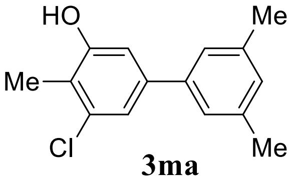

3ma

A mixture of PEPPSI-IPr (8.5 mg, 0.0125 mmol), Ag_2CO_3 (69.0 mg, 0.25 mmol), K_2CO_3 (69.0 mg, 0.50 mmol), 4-chloro-2-hydroxy-3-methylbenzaldehyde (42.6 mg, 0.25 mmol) and 1-iodo-3,5-dimethylbenzene (110 µL, 0.75 mmol) in AcOH (500 µL) was heated at 150 ºC for 16 h. After this time, the reaction mixture was filtered through a plug of Celite® with EtOAc (4 × 5 mL). The filtrate was evaporated to dryness. The crude product was purified by long column chromatography (Hexanes:EtOAc 50:50) to afford 5-chloro-3',4,5'-trimethyl-[1,1'-biphenyl]-3-ol (**3ma**) as a colorless oil (32.7 mg, 53%). **IR**: 3286, 2919, 1568, 1390, 1265, 1144, 1019, 810 cm^{-1}. **^{1}H NMR** (400 MHz, $CDCl_3$) δ 7.20 (app. d, J = 1.6 Hz, 1H), 7.14 (s, 2H), 7.00 (m, 1H), 6.90 (app. d, J = 1.6 Hz, 1 H), 4.88 (s, 1H), 2.37 (s, 6H), 2.33 (s, 3H) ppm. **^{13}C NMR** (101 MHz, $CDCl_3$) δ 154.6, 140.7, 139.5, 138.4,

135.6, 129.4, 124.7, 121.3, 120.4, 112.2, 21.4, 12.4 ppm. These data are consistent with those previously reported.[3]

References

[1] N. U. Hofsløkken and L. Skattebøl, *Acta Chemica Scandinavica*, **1999**, *53*, 258.
[2] D. H. T. Phan, B. Kim and V. M. Dong, *J. Am. Chem. Soc*., **2009**, *131*, 15608.
[3] J. Luo, S. Preciado and I. Larrosa, *J. Am. Chem. Soc*., **2014**, *136*, 4109.
[4] J. Luo, S. Preciado and I. Larrosa, *Chem. Commun*., **2015**, *51*, 3127.
[5] H. Rao, C.-J. Li, *Angew. Chem. Int. Ed*., **2011**, *50*, 8936.
[6] Y. Izawa, C. Zheng, and S. S. Stahl, *Angew. Chem. Int. Ed*., **2013**, *52*, 3672.

Spectroscopic data

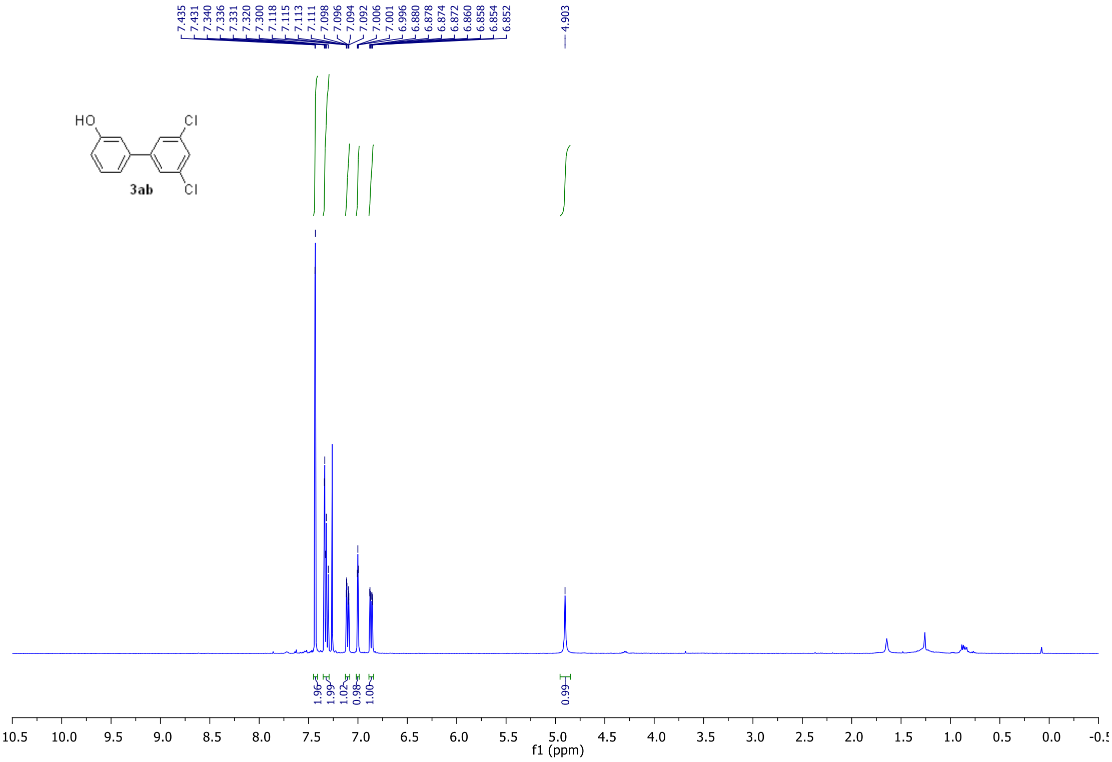

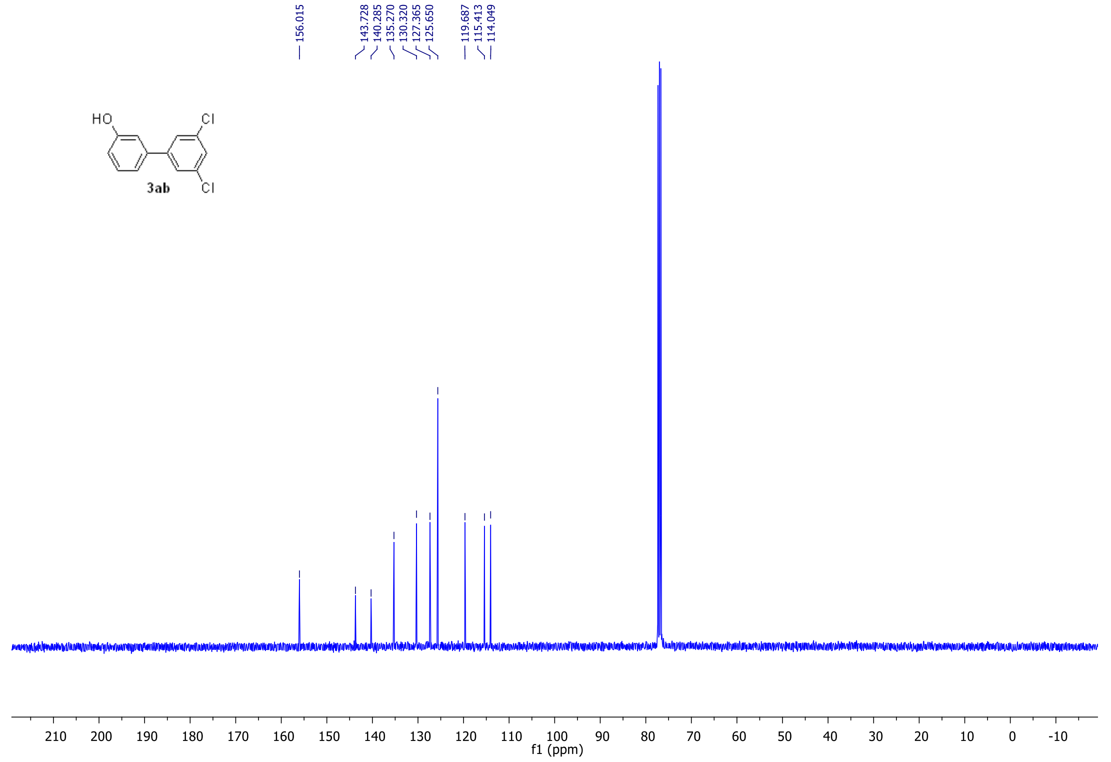

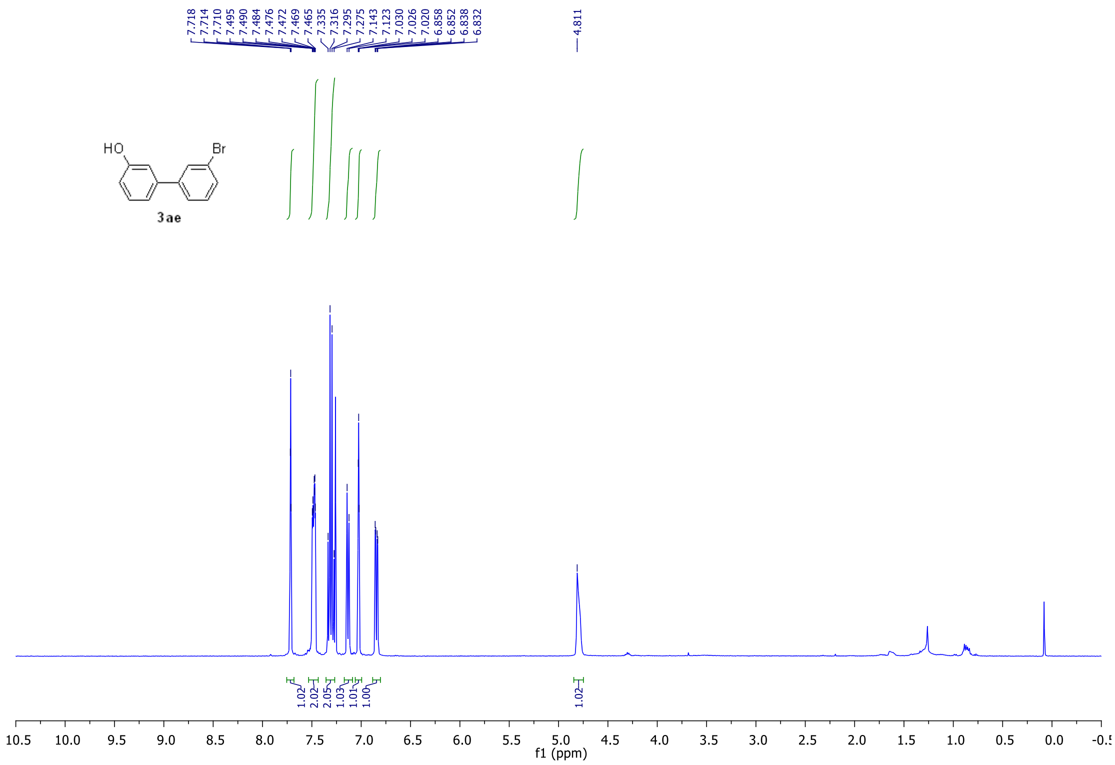
HO
Br
3ae
7.718
7.714
7.710
7.495
7.490
7.484
7.476
7.472
7.469
7.465
7.335
7.316
7.295
7.275
7.143
7.123
7.030
7.026
7.020
6.858
6.852
6.838
6.832
4.811
1.02
2.02
2.05
1.03
1.01
1.00
1.02
f1 (ppm)

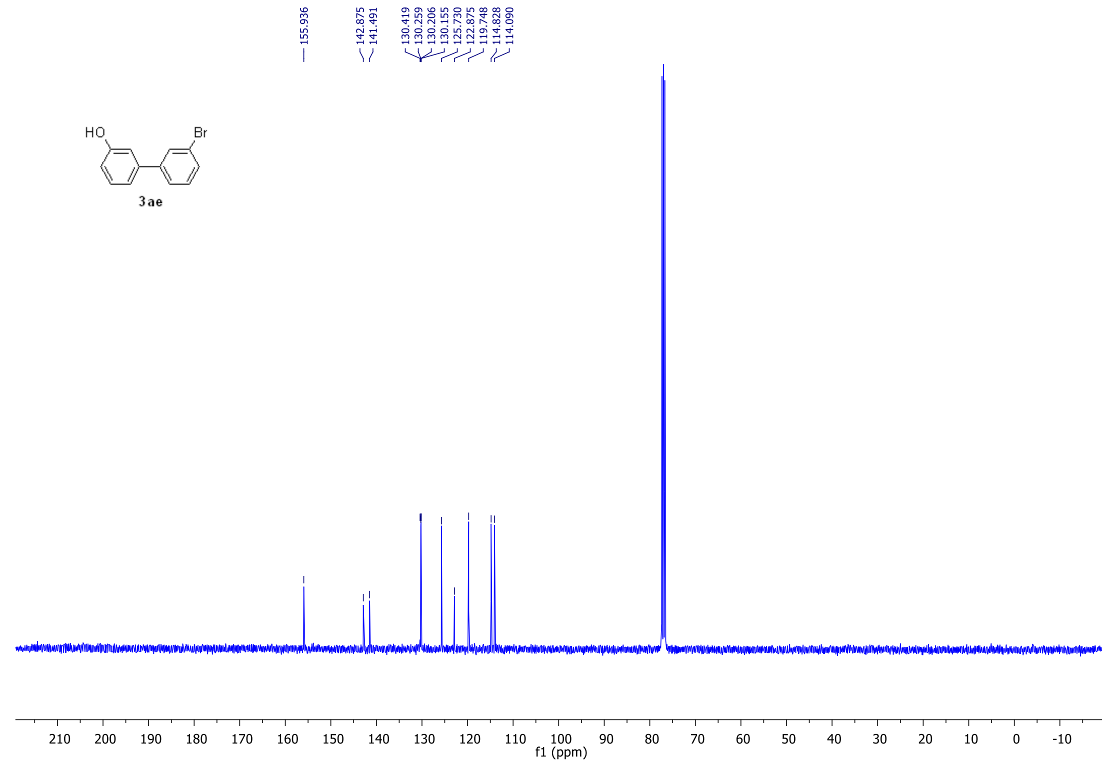
HO
Br
3ae
155.936
142.875
141.491
130.419
130.259
130.206
130.155
125.730
122.875
119.748
114.828
114.090
f1 (ppm)

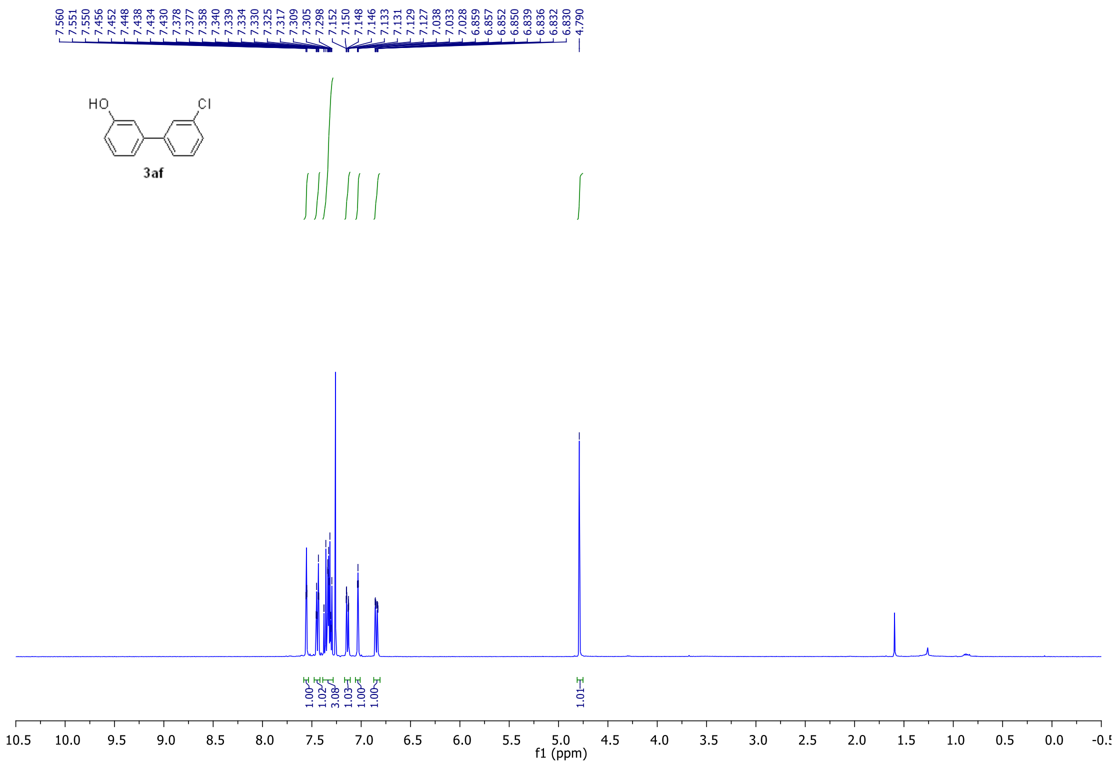

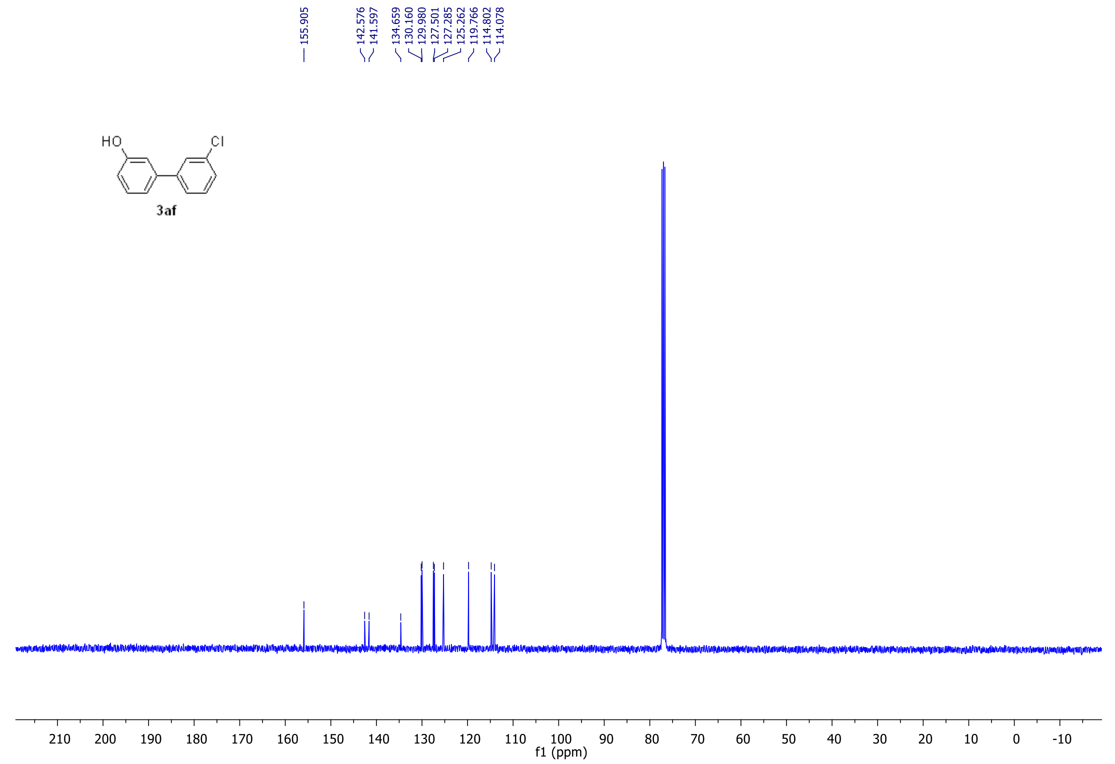

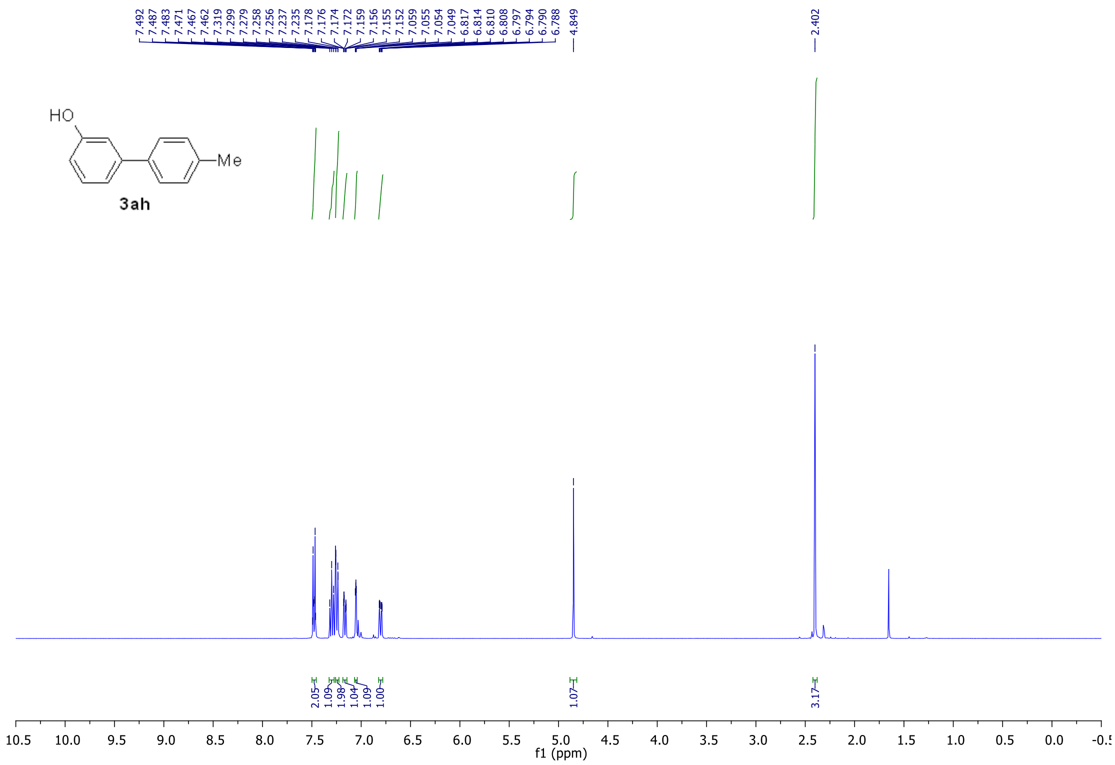
HO
Me
3ah
f1 (ppm)

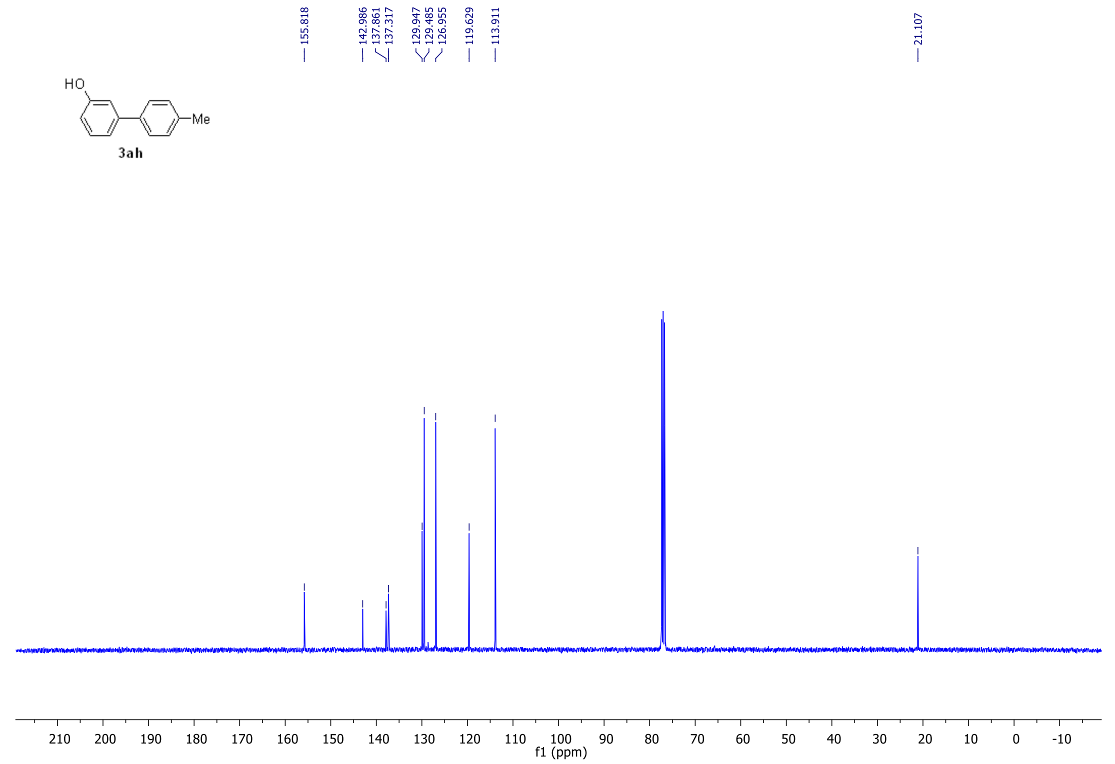
HO
Me
3ah
f1 (ppm)

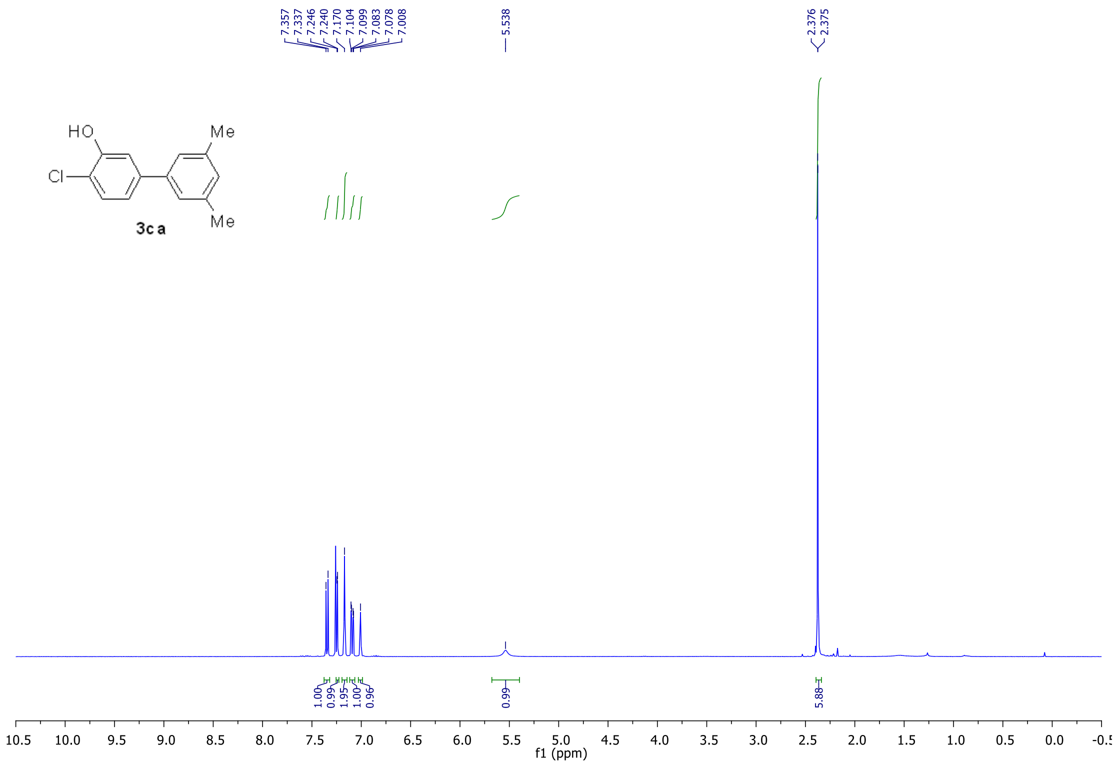
HO
Cl
Me
Me
3ca
7.357
7.337
7.246
7.240
7.170
7.104
7.099
7.083
7.078
7.008
5.538
2.376
2.375
1.00
0.99
1.95
1.00
0.96
0.99
5.88
f1 (ppm)

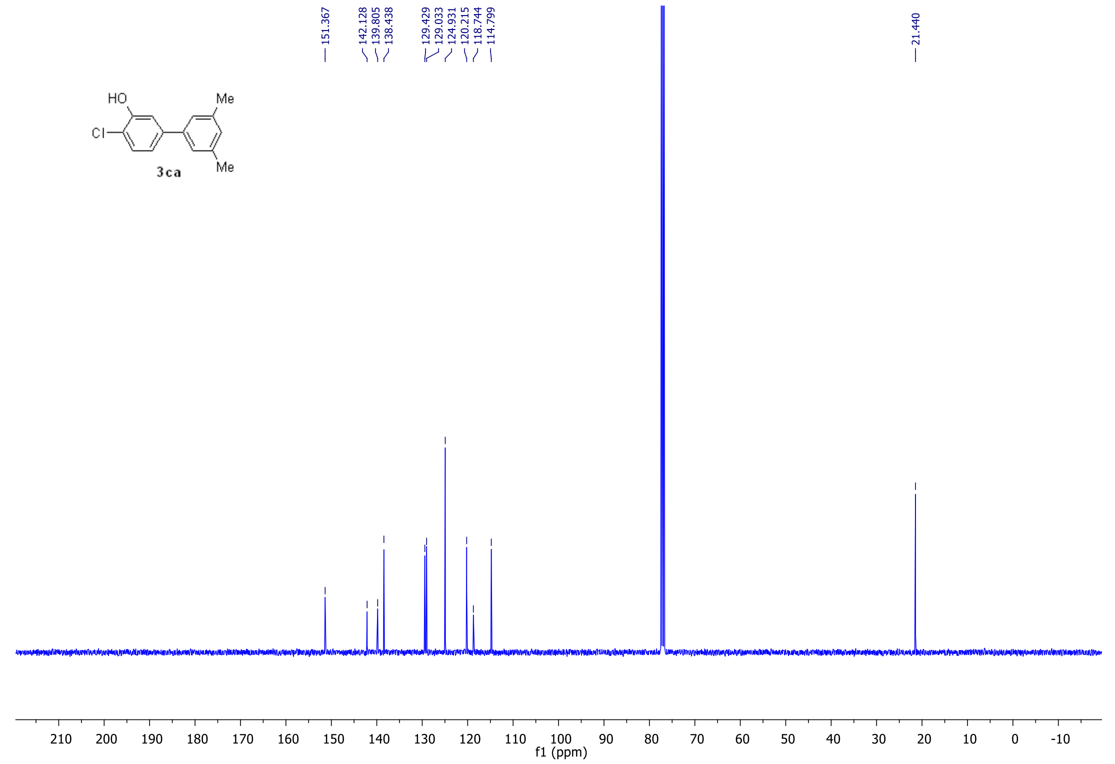
HO
Cl
Me
Me
3ca
151.367
142.128
139.805
138.438
129.429
129.033
124.931
120.215
118.744
114.799
21.440
f1 (ppm)

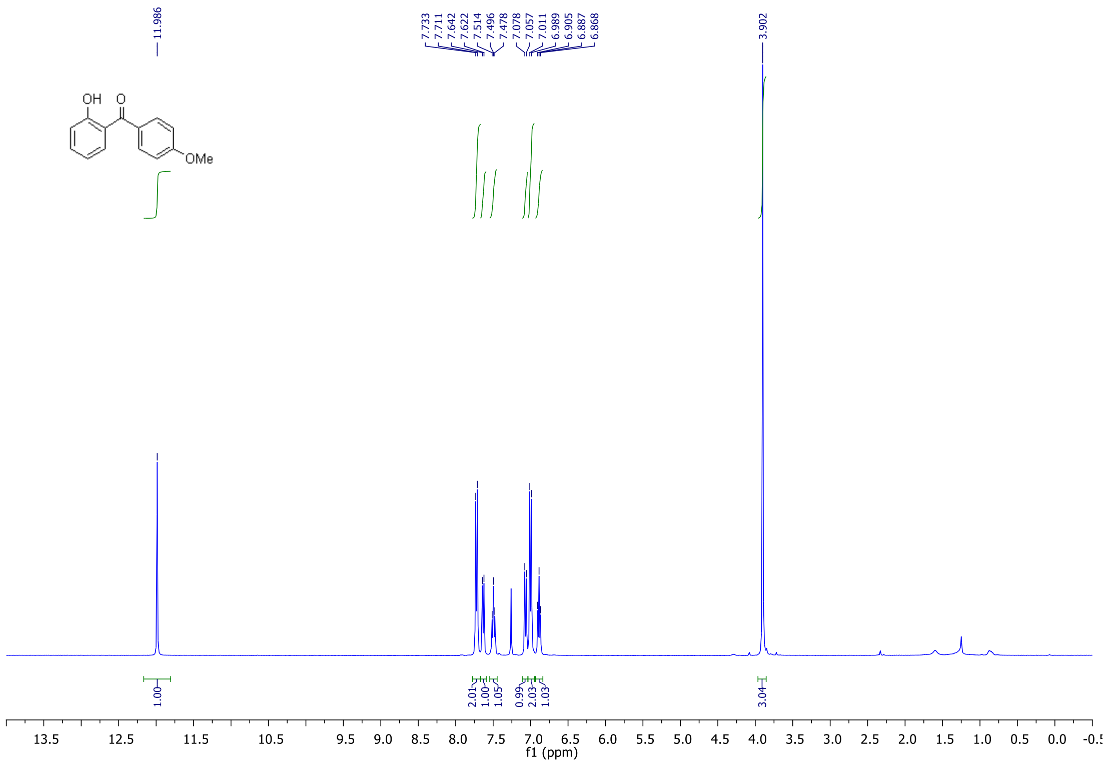
11.986
7.733
7.711
7.642
7.622
7.514
7.496
7.478
7.078
7.057
7.011
6.989
6.905
6.887
6.868
3.902
OH
O
OMe
1.00
2.01
1.00
1.05
0.99
2.03
1.03
3.04
f1 (ppm)

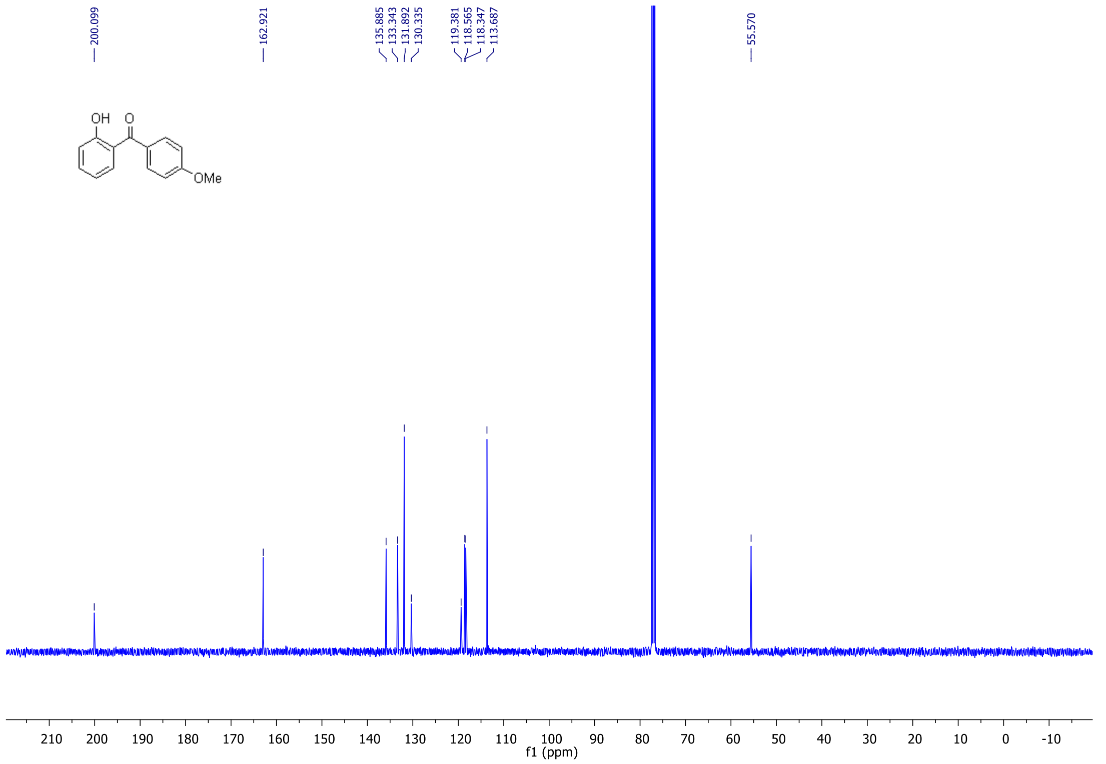
200.099
162.921
135.885
133.343
131.892
130.335
119.381
118.565
118.347
113.687
55.570
OH
O
OMe
f1 (ppm)
